# Analysis of hydrazine in smokeless tobacco products by gas chromatography–mass spectrometry

**DOI:** 10.1186/s13065-015-0089-0

**Published:** 2015-03-16

**Authors:** Kevin McAdam, Harriet Kimpton, Sofia Essen, Peter Davis, Carl Vas, Christopher Wright, Andrew Porter, Brad Rodu

**Affiliations:** Group Research & Development, British American Tobacco, Regents Park Road, Southampton, SO15 8TL UK; 3810 St Antoine W, Montreal, QC H4C 1B4 Canada; Room 208, 505 South Hancock Street, Louisville, KY 40202 USA

**Keywords:** Hydrazine, Smokeless tobacco products, Snus, Snuff

## Abstract

**Background:**

Due to the lower health risks associated with the use of certain categories of smokeless tobacco products (STPs) such as Swedish snus, there is interest in the comparative levels of toxic chemical constituents in different types of STPs. A method has been developed and validated for the analysis of hydrazine in STPs. Seventy four commercial STPs from the US and Sweden, representing 80-90% of the 2010 market share for all the major STP categories in these two countries, as well as three reference STPs, were analysed for hydrazine.

**Results:**

Aqueous extracts of the STPs were treated with excess pentafluorobenzaldehyde (PFB), which reacted with hydrazine in solution to form decafluorobenzaldehyde azine (DFBA). DFBA was partitioned into hexane and then quantified by gas chromatography–mass spectrometry (GC–MS). The method was validated using five different types of STP, was linear in the range 8–170 ng/mL, and had limits of quantification (LOQ) from 26–53 ng of hydrazine per g of STP (as sold). The method was applied to the analysis of 74 contemporary STPs commercially available in the United States and Sweden, none of which were found to contain hydrazine above the LOQ or LOD. Trace levels of compounds showing chromatographic and mass spectral features consistent with hydrazine were identified at very low levels (sub-limit of detection, <10 ng/g) in the chromatograms of less than half of the 74 STPs examined; in contrast, for 40 of the STPs no evidence for the presence of hydrazine was observed. Where present, the levels of compounds consistent with hydrazine were estimated to be at least an order of magnitude lower than the only previous study to have quantified hydrazine in tobacco.

**Conclusions:**

Our results show that hydrazine is not a prevalent constituent of STPs, and when present is not quantifiable using currently available analytical methodology.

## Background

Smokeless Tobacco Products (STPs) have been collectively designated as Group 1 carcinogens i.e. carcinogenic to humans [1], but there is considerable evidence that health risks differ between STP categories, with certain product styles such as Swedish snus having lower health risks associated with their use [2]. As a result, there is substantial interest in the comparative levels of toxic chemical constituents of the different types of STPs. In the US, the Food and Drug Administration (FDA) has assembled a list (“The Established List”) of 93 “hazardous or potentially hazardous constituents” (HPHC) of tobacco products which may have to be reported [3]. This list covers both tobacco and tobacco smoke components and includes 79 that are designated as carcinogenic as well as constituents that are respiratory toxicants, cardiovascular toxicants, reproductive toxicants or addictive. One of the HPHC carcinogens on the list is hydrazine (N_2_H_4_) which has been classified as a group 2B carcinogen (possibly carcinogenic to humans) by IARC [4].

Although studies of hydrazine toxicity in humans are limited, human exposure to hydrazine has resulted in severe effects on the central nervous system, liver and kidneys [4]. Hydrazine is mainly an industrial chemical, manufactured from ammonia, that can enter the environment from facilities that manufacture, process or use it. Hydrazine is unstable and degrades rapidly in most environmental media. It can dissolve in water and move though soil, but hydrazine is broken down by autoxidation and by microorganisms. A review [5] of three studies concluded that half-lives of hydrazine in soil ranged from 1 hr to 3 days with the more rapid degradation of hydrazine occurring in soils with high levels of microorganisms and organic material.

The occurrence of free hydrazines in nature is rare. Naturally occurring hydrazine and hydrazone derivatives such as agaritine (β-N-[γ-L(+)-glutamyl]-4-hydroxymethyl phenylhydrazine) and gyromitrin (acetaldehyde methylformylhydrazone) have been reported in mushrooms [6]. Gyromitrin breaks down during cooking to release methylhydrazine but the latter is not found in a free state in the mushroom. Hydrazine is produced as an intermediate during biological nitrogen fixation by the molybdenum- and vanadium-based nitrogenase enzymes in *Azotobacter* [7]. In the case of the more abundant molybdenum-based nitrogenase the hydrazine is bound to the enzyme and is not released in a free state. However for vanadium- based nitrogenase small but significant amounts of free hydrazine are generated [8]. There is therefore the potential for hydrazine to be found in plant materials that are associated with nitrogenase containing bacteria.

The only occurrence of free hydrazine in plant material was that reported in tobacco by Liu et al. [9]. They found small amounts of hydrazine in tobacco from a commercial cigarette (30.0 ng/cigarette) and in the tobacco of four experimental cigarettes. Two of the experimental cigarettes were made with Burley tobaccos, one treated with the plant sucker growth inhibitor maleic hydrazide (MH) (51.2 ng hydrazine/cigarette) and one untreated (22.2 ng hydrazine/cigarette); the other two cigarettes were made with flue-cured tobacco, one treated (12.1 ng hydrazine/cigarette) and one untreated (13.8 ng hydrazine/cigarette). Liu et al. [9] also determined hydrazine in the mainstream smoke of these 5 cigarettes (range 23.5–42.8 ng/cigarette). The hydrazine concentrations in tobacco and tobacco smoke obtained in the original Liu et al. study of over 40 years ago [9], have been frequently reproduced in review articles [10-16]. No other study of hydrazine in tobacco has been reported, although several other studies have failed to detect hydrazine in tobacco smoke [17-19]. Using the same methodology as Liu et al. for hydrazine, Schmeltz et al. [13] found the hydrazine derivative, 1,1-dimethylhydrazine, in several samples of tobacco including US chewing tobacco (97.7 ng/g) and snuff (96.7 ng/g), four commercial US cigarette blends (60.2 ± 5.7 ng/g) and Bright tobacco (147 ng/g). No 1,1-dimethylhydrazine was found in a sample of Burley tobacco.

We are currently conducting a comprehensive survey of toxicants in an extensive and varied set of contemporary STPs from the United States and Sweden. There have been no further published studies of hydrazine in tobacco since the report by Liu et al. in 1974, and no studies of hydrazine in STPs have ever been reported. The aims of the present study were therefore to develop and validate a method for the analysis of hydrazine in STPs, and to survey major STPs from the USA and Sweden for their hydrazine content.

**Figure 1:**
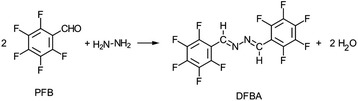
**Reaction of pentafluorobenzaldehyde (PFB) with hydrazine to form decafluorobenzaldehyde azine (DFBA).**

Various methods have been reported for the detection of trace levels of hydrazine in substrates such as sludge, human plasma, environmental water and drug samples. These have included chemical derivatization with reagents such as benzaldehyde, 4-hydroxy benzaldehyde, 2-hydroxynaphthaldehyde, 2,4-dinitrochlorobenzene and acetone or acetone-d6, coupled with HPLC and/or spectrophotometric detection [20-23], solid-phase spectrophotometry [24], and GC–MS [25]. Indirect methods have also been developed, such as oxidation of hydrazine by excess iodate [26] or bromine [27], and analysis of the unreacted oxidant. The detection limits for these methods vary from 1 ng/ml [21] to 100 ng/g [25]. The analytical method used in the original study in tobacco by Liu et al. [9] was based on the reaction of residues of hydrazine in tobacco product extract with PFB to form DFBA, Figure 1, coupled with multiple thin-layer chromatographic steps, followed by GC separation and electron capture detection.

For the analysis of STPs, we chose to use derivatization of hydrazine with PFB, as described in the Liu et al. paper, but coupled with GC–MS to improve the sensitivity and to eliminate the need for the multiple concentration steps used in the original paper. The present method was based on the reaction of residues of hydrazine in tobacco product extract with PFB to form DFBA. The DFBA is partitioned into hexane and then quantified by GC–MS. After validation of this approach, the method was applied to the analysis of 74 contemporary STPs commercially available in the United States and Sweden. The products covered all major STP categories and the brands selected represented 90% market share of the major product styles [28].

## Results and discussion

### Validation of the analytical method

As a first step in validating the analytical approach, the identity of the peak assigned to DFBA was confirmed by visual examination of the chromatograms and mass spectra obtained for standards and tobacco samples spiked with hydrazine at 0.53 μg/g. The retention time of the GC peak assigned to DFBA was 9.9 min for all standards and types of STP. The mass spectra of the assigned peaks were almost identical for all standards and spiked tobacco samples and included ion clusters at m/z 388 (molecular ion and base peak), 194, 180, 117 and 93. Lastly, the MS software selected the spectrum of DFBA from the “Saturn library” as the closest match to that of the chromatogram peak.

**Figure 2:**
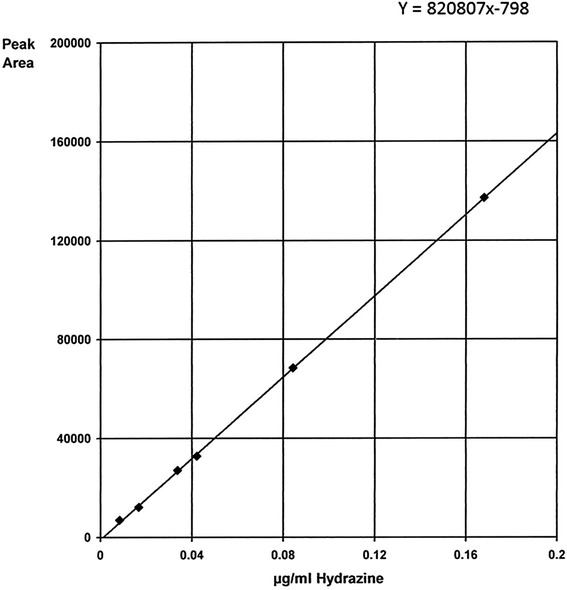
**Linearity of the analytical method.** The intercept on the *x* axis represents a hydrazine concentration of 0.0018 μg/mL.

To check the linearity of the method, six standard DFBA solutions, ranging from 100 to 2000 ng/mL (equivalent to 8–170 ng/mL hydrazine), were applied to GC–MS in a random order. The areas of the peaks were subjected to linear regression analysis. The analysis was highly linear across the six standards with *R*^2^ values in excess of 0.99 (Figure 2).

In a preliminary analysis, the five control tobacco samples (Garrett, Stonewall Wintergreen, Ettan Loose, Days Work and Taylor’s Pride), each representing a different type of STP, were analysed in duplicate. All of these STPs had apparent hydrazine contents below the lowest standard analysed (100 ng/mL DFBA), corresponding to concentrations of <16 ng/g.

To test the accuracy and precision of the analytical method, analyses were carried out in which the five control STPs were spiked with hydrazine at 530 ng/g, 53 ng/g and 26.5 ng/g. For each STP at each level of spiking, five repeat analyses were carried out (Table 1).

**Table 1:** **Recovery and repeatability of measurements of spiked tobacco samples**

**Sample**	**Style**	**Hydrazine spike level (ng/g)**	**Percentage recovery, mean ± RSD (n = 5)**	**Overall percentage RSD**
Garrett	Dry snuff	530	87.5 ± 5.1	
		53.0	105.2 ± 4.7	10.7
		26.5	96.7 ± 11.9	
Stonewall Wintergreen	Pellet	530	77.0 ± 5.0	
		53.0	74.1 ± 6.9	10.8
		26.5	64.0 ± 11.3	
Ettan	Loose snus	530	75.7 ± 8.3	
		53.0	96.1 ± 6.9	11.9
		26.5	83.1 ± 3.3	
Days Work	Plug	530	85.2 ± 4.1	
		53.0	95.3 ± 8.5	7.8
		26.5	92.2 ± 6.2	
Taylor’s Pride	Chewing tobacco	530	90.5 ± 12.0	
		53.0	98.3 ± 3.2	9.15
		26.5	99.7 ± 9.1	

At spike levels of 530 and 53 ng/g hydrazine, the mean recoveries from all tobacco product types were within the acceptable range [29] of 70%–110% (Table 1), indicating satisfactory accuracy. At the spike level of 26.5 ng/g hydrazine, a low recovery of 64% was observed for Stonewall Wintergreen. The hard pellet has a high mineral content that may have affected the hydrazine added, either through chemical reaction or adsorption. With the %RSD below 20%, the precision of the analytical technique was satisfactory at all three spike levels.

The LOQ was defined as the lowest spike level for which there was an acceptable recovery (i.e. in the range 70%-110%). The LOQ was therefore 26.5 ng/g for the chewing tobacco, dry snuff, moist snuff and loose snus, and 53 ng/g for the hard pellet. The limit of detection (LOD) was estimated to be less than 10 ng/g from this validation. The linearity, LOQ, LOD, precision, and accuracy of the method are summarized in Table 2.

**Table 2:** **Linearity and sensitivity of the analytical method for hydrazine**

**Validation parameter**	**Value**
Linear range	8–170 ng/mL
Linear curve	*y* = 1.03 × 10^6^ *x* − 1886.88
*R* ^2^	0.9996
Accuracy across 5 types of STP (% mean ± RSD recovery)	64.0%–105.2%
Accuracy across 4 types of STP^a^ (% mean ± RSD recovery)	75.7%–105.2%
Precision across 5 types of STP (%RSD of repeatability of spike recovery)	7.8%–11.9%
LOQ for 5 types of STP	53.0 (ng/g)
LOQ for 4 types of STP^a^	26.5 (ng/g)
LOD	<10 (ng/g)

^a^Without the pellet product, which showed poor recovery.

### Survey of 74 STPs for hydrazine

**Figure 3:**
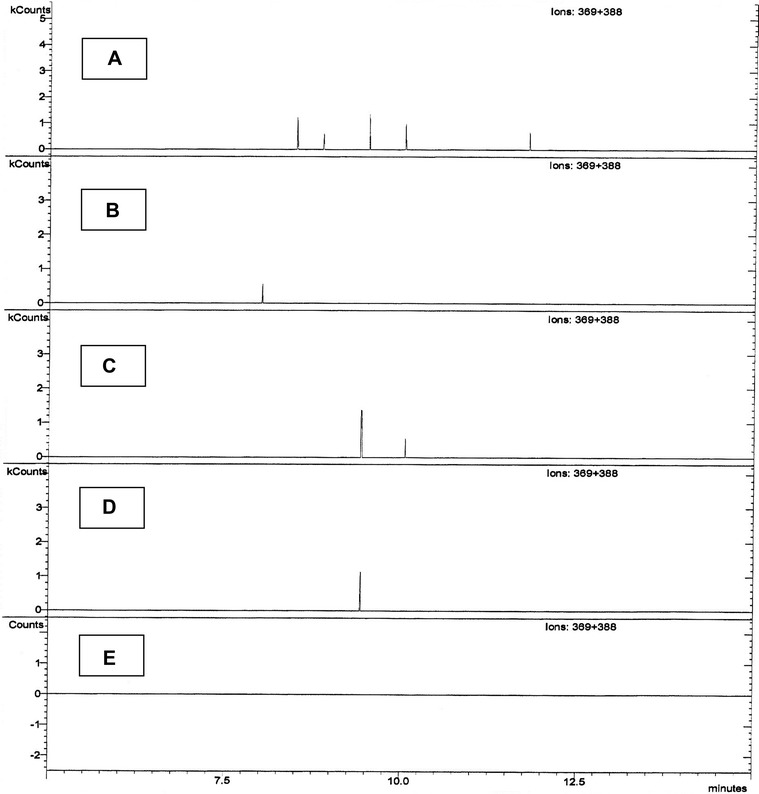
**Typical chromatograms for smokeless tobacco products in the analysis of hydrazine (retention time 10.0 minutes); A) Camel Frost Snuff, B) Camel Mellow Snuff C) Marlboro Mild Snuff, D) Marlboro Peppermint Snuff, E) Marlboro Rich Snuff.**

Once validated, the method was used to survey the 74 contemporary STPs for hydrazine levels. As shown in Tables 3 and 4, none of the products was found to contain hydrazine at levels above the LOD (<10 ng/g). Examination of the chromatograms identified a very low level peak at the retention time of hydrazine (Figure 3A, C), and with a matching mass spectrum, for 34 of the 74 STPs analysed. The peaks were sufficiently infrequent, and below the LOD, that the possibility cannot be discounted that these peaks were merely analytical noise. However, as the peaks showed identical chromatographic and mass spectral features to hydrazine, we regard it as possible that very low levels of hydrazine were present in the samples showing these peaks. Of these STPs, the majority (22) showed the hydrazine peak in only one of the three replicates analysed, seven STPs showed the peak in two of the three replicates, and five STPs showed the peak in all three replicates. In total 51 of the 222 replicate analyses showed the presence of hydrazine, and therefore the majority of analyses showed no evidence for the presence of low levels of hydrazine (Figure 3B, D, E). Clearly, if there is interest in quantifying these potential very low levels of hydrazine, a much more sensitive analytical method would be required, with more than an order of magnitude greater sensitivity. It is unlikely that the current approach is modifiable to this extent, and alternative approaches may be required.

**Table 3:** **Estimated hydrazine concentrations in Swedish STPs**

**Swedish STPs**	**Style**	Hydrazine content (ng/g)**	**No of replicates with peaks below LOD corresponding to hydrazine**
Ettan	Loose snus	<LOD	1
General		<LOD	0
Goteborgs Rape		<LOD	0
Granit		<LOD	0
Grovsnus		<LOD	3
Knox		<LOD	0
Kronan		<LOD	0
LD Original		<LOD	0
T. Montecristo		<LOD	0
Skruf Strong		<LOD	1
Catch Licorice, mini	Portion snus	<LOD	0
Catch White Licorice		<LOD	2
CatchDry White Eucalyptus, mini		<LOD	1
Ettan		<LOD	0
General		<LOD	0
General mini		<LOD	0
General White		<LOD	0
Goteborgs Rape		<LOD	0
Granit		<LOD	1
Granit White		<LOD	0
Grovsnus		<LOD	0
Grovsnus White		<LOD	0
Gustavus Original		<LOD	1
Knox		<LOD	0
Kronan		<LOD	0
LD Original		<LOD	0
Oomph Citrus Menthol		<LOD	1
Romeo y Julieta Habanos		<LOD	0
Skruf Strong		<LOD	1
Tre-Ankare White		<LOD	1
1847 Original		<LOD	0
CRP1		<LOD	0

All STPs have mean hydrazine contents below the LOD (<10 ng/g STP “as sold”*).

*All hydrazine concentrations are based on the “as sold” or wet weight of STP, with no corrections for moisture.

**Table 4:** **Estimated hydrazine concentrations in US STPs**

**US STPs**	**Style**	**Hydrazine content (ng/g)**	**No of replicates with peaks below LOD corresponding to hydrazine**
Beech Nut	Chewing tobacco	<LOD	2
Chattanooga		<LOD	2
Durango		<LOD	0
Lancaster		<LOD	0
Levi Garrett		<LOD	1
Morgans		<LOD	1
Red Man Gold		<LOD	0
Red Man Regular		<LOD	0
Southern Pride		<LOD	0
Starr		<LOD	1
Stoker 707 Wintergreen		<LOD	0
Taylors Pride		<LOD	0
Trophy		<LOD	1
Bruton	Dry snuff	<LOD	2
Dental Sweet		<LOD	0
Garrett		<LOD	3
Honest		<LOD	3
Square		<LOD	1
CRP3		<LOD	0
Ariva Java	Hard pellet	<LOD	1
Stonewall Wintergreen		<LOD	1
Oliver Twist Original	Soft pellet	<LOD	1
Copenhagen LC	Moist snuff	<LOD	-
Copenhagen Straight LC		<LOD	0
Grizzly Natural LC		<LOD	0
Husky Natural FC		<LOD	1
Husky Straight LC		<LOD	2
Husky Wintergreen		<LOD	3
Kayak Straight LC		<LOD	3
Kodiak Straight LC		<LOD	1
Kodiak Wintergreen		<LOD	0
Red Seal Natural FC		<LOD	1
Red Seal Natural LC		<LOD	1
Silver Creek		<LOD	2
Skoal Straight		<LOD	1
Timber Wolf Natural FC		<LOD	0
Timber Wolf Straight LC		<LOD	0
CRP2		<LOD	2
Cannonball	Plug	<LOD	-
Camel Frost	US snus	<LOD	1
Camel Mellow		<LOD	0
Marlboro Mild		<LOD	1
Marlboro Peppermint		<LOD	0
Marlboro Rich		<LOD	0
Marlboro Spearmint		<LOD	1

All STPs have mean hydrazine contents below the LOD (<10 ng/g STP “as sold”).

As noted above, in every case that hydrazine was tentatively identified in the current study the peak areas were substantially below the LOQ and LOD of the current method, and therefore the levels present cannot be determined. However, overall, our results indicate that hydrazine is not a prevalent contaminant of contemporary STPs, and in the minority of cases where a peak consistent with hydrazine was observed, the levels present are substantially lower than those reported previously by Liu et al. [9].

### Sources of hydrazine in tobacco

Liu et al. [9] considered the possibility that the MH used as a sucker growth inhibitor on the tobacco crop was the source of hydrazine observed in their tobacco. Hydrazine is a contaminant in MH that derives partly from the manufacturing process and partly from subsequent breakdown of MH (particularly the formulation conjugated with diethanolamine, MH-30) [30]. However Liu et al. [9], albeit on a limited number of samples, found no relationship between MH concentrations and hydrazine. MH treated samples of tobacco had similar levels of hydrazine to samples containing no MH. Subsequent to the Liu et al. study, the diethanolamine salt was banned (in 1980) and only the more stable potassium salt of MH is currently approved for use. The US EPA [31] and the European Union [32] have also introduced limits on the concentration of hydrazine in MH - 15 ppm in the US and 1 ppm (1 μg/g) in the EU. Using the CORESTA issued Guidance Residue Levels (GRL) on agrochemicals of 80 ppm for MH on tobacco [33] as an upper limit, and assuming no hydrazine losses from the tobacco post MH-application, it can be calculated that maximum concentrations of hydrazine in tobacco arising from contaminated MH would be 1.2 ng/g in the US and 0.08 ng/g in the EU. The current study therefore does not rule out the possibility that breakdown of MH might contribute to traces of hydrazine in the tobacco.

An alternative to MH as a source of hydrazine in tobacco was advanced by Schmeltz et al. [13]. They reported the hydrazine derivative, 1,1-dimethylhydrazine, in several samples of US tobaccos using the same methodology as Liu et al. [9]. Schmeltz et al. [13] proposed that unspecified bacterial and enzymatic processes that occur during curing might be responsible for producing both the 1,1-dimethylhydrazine observed in their study and also the hydrazine observed in the earlier Liu et al. study. To date, however, there have been no reports of microorganisms or enzymatic pathways, such as nitrogenase, specifically related to tobacco that would result in hydrazone or hydrazine formation.

### Differences between levels reported by Liu and results of the present study

**Figure 4:**
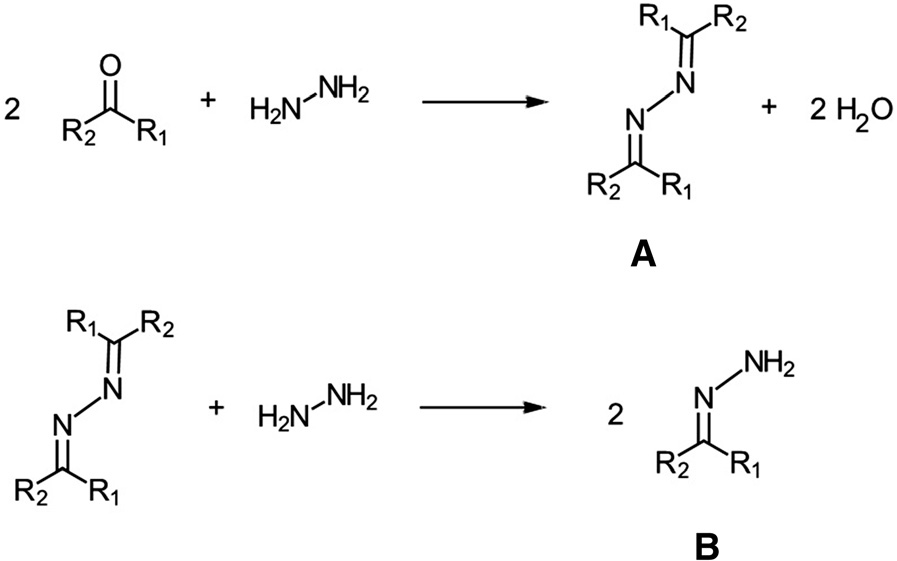
**Reaction of ketones (R**_**1**_ **, R**_**2**_ **= alkyl) and aldehydes (R**_**1**_ **= alkyl, R**_**2**_ **= H) with hydrazine to form azines (A) and hydrazones (B).**

Whatever the source of the hydrazine it is unlikely that it would be present in tobacco in a free state. Hydrazine is a powerful reducing agent, and reacts with carbonyls to form azines and hydrazones as shown in Figure 4 [34]. STPs and other forms of tobacco have been shown to contain ppm levels of formaldehyde, acetaldehyde, crotonaldehyde [10,35,36], acrolein [36], acetone, propionaldehyde, isobutyraldehyde, 2-butanone isovaleraldehyde and valeraldehyde [35]. Although the presence of carbonyl azines or hydrazones in tobacco has not been reported [37], given the thousand-fold excess of carbonyls over hydrazine in tobacco, there is a strong possibility that any hydrazine present in the tobacco plant will react with the carbonyls present. The observation that hydrazine reacts rapidly with acetaldehyde in tobacco smoke [38] adds to the plausibility that the same reaction can occur in the tobacco matrix. Moreover hydrazine is relatively volatile with a boiling point of 114°C and it would seem probable that any unreacted hydrazine would be lost during processing particularly for STPs such as snus where there is sustained heat treatment of the tobacco.

**Figure 5:**
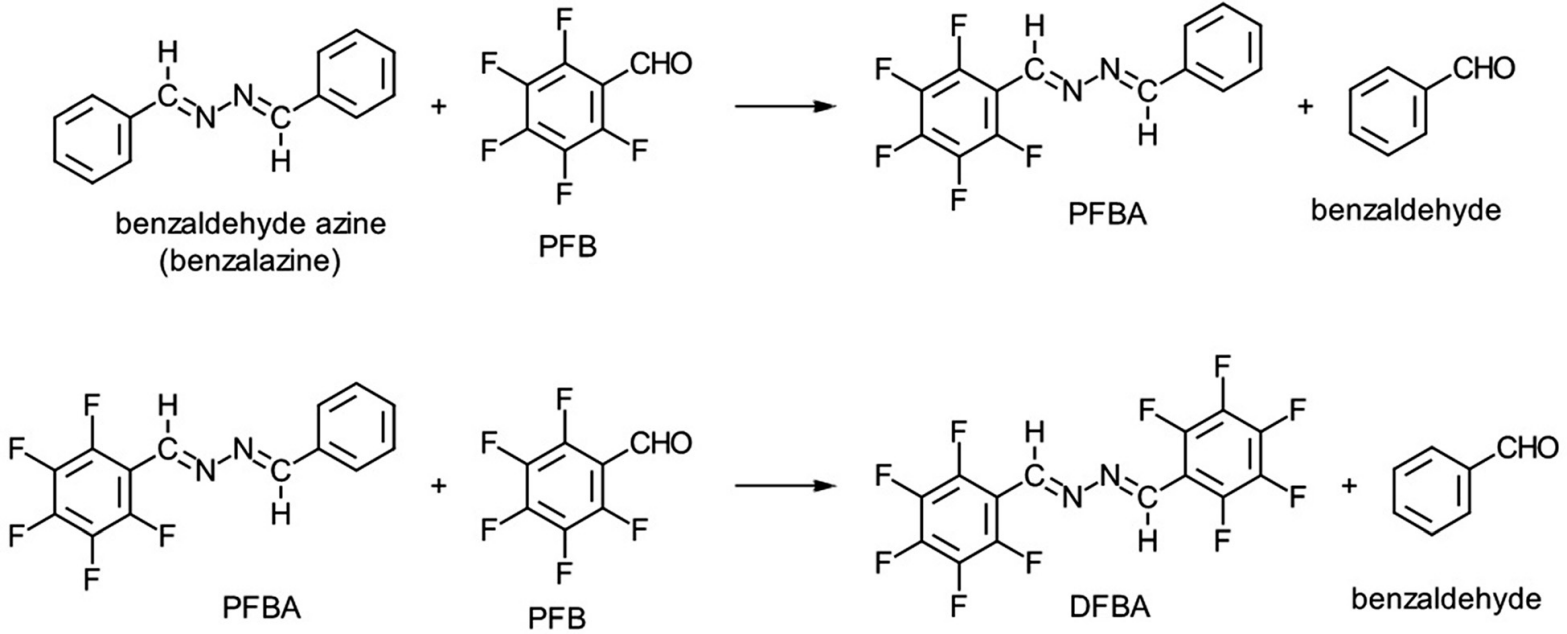
**Reaction of benzaldehyde azine (benzalazine) with pentafluorobenzaldehyde (PFB) to form pentafluorobenzaldehyde azine (PFBA) and decafluorobenzaldehyde azine (DFBA).**

Liu et al. [9] pointed out that the highly reactive complexing agent PFB not only reacts with any free hydrazine in the matrix but is also able to react with any hydrazones or azines that may be present. They demonstrated this by showing that more than 70% of the hydrazine moiety of benzalazine was detected as pentafluorobenzaldehyde azine (PFBA) during controlled experiments over a 16 hour period representative of their experimental extraction conditions for tobacco and smoke (Figure 5). Thus we would expect that any hydrazones and azines present in the tobacco matrix could also react with PFB. Hence the hydrazine content of tobacco reported by Liu et al. may well have been the sum of hydrazine, hydrazones and azines present.

In the present study a much shorter contact time between tobacco and the reactive complexing agent PFB was used. The one hour complexation time used in the present study is an order of magnitude shorter than the “overnight” time used by Liu et al. [9]. Notably, the hydrazine contents identified in this work are an order of magnitude lower than reported by Liu et al. [9]. One explanation for the difference in contents could therefore be a restricted opportunity for reaction of PFB with hydrazones or azines in this work compared to that in the study of Liu et al. [9].

### Experimental

#### Tobacco samples

Tobacco samples were obtained in 2010. Details of the STP markets in the United States and Sweden were obtained, and the products for analysis were chosen to reflect approximately 90% market share of the major STP categories in these two markets at that time. The major products in each category of STP were sampled. In total, the survey comprised 31 Swedish products (10 loose snus and 21 portion snus, Table 5) and 43 US products (13 chewing tobaccos, 5 dry snuffs, 2 hard pellet products, 1 soft pellet product, 15 moist snuffs, 6 US snus and 1 plug product, Table 6). The Swedish products were sourced from Swedish retail websites, imported into the United Kingdom, and kept frozen at −20°C until analysis. The US products were sourced from shops in the United States, imported, and kept frozen at −20°C until analysis. Three CORESTA reference STP products [39] were also sampled and analysed in this exercise, CRP1 (Swedish snus pouch product), CRP2 (US style loose moist snuff) and CRP3 (US style loose dry snuff powder).

**Table 5:** **Swedish STPs**

**Swedish STPs**	**Style**	**Manufacturer**	**Water content (%)**
Ettan	Loose snus	Swedish Match	46.7
General		Swedish Match	46.4
Goteborgs Rape		Swedish Match	48.5
Granit		Fiedler & Lundgren	44.8
Grovsnus		Swedish Match	46.4
Knox		Swedish Match	43.4
Kronan		Swedish Match	48.5
LD Original		Japan Tobacco Co.	48.0
T. Montecristo		Habanos Nordics	49.7
Skruf Strong		Skruf	49.0
Catch Licorice, mini	Portion snus	Swedish Match	42.4
Catch White Licorice		Swedish Match	46.5
CatchDry White Eucalyptus, mini		Swedish Match	22.4
Ettan		Swedish Match	42.3
General		Swedish Match	41.3
General mini		Swedish Match	44.1
General White		Swedish Match	45.3
Goteborgs Rape		Swedish Match	45.1
Granit		Fiedler & Lundgren	43.2
Granit White		Fiedler & Lundgren	39.1
Grovsnus		Swedish Match	43.9
Grovsnus White		Swedish Match	45.3
Gustavus Original		Japan Tobacco Co.	43.0
Knox		Swedish Match	40.6
Kronan		Swedish Match	43.2
LD Original		Japan Tobacco Co.	44.0
Oomph Citrus Menthol		Northerner	4.2
Romeo y Julieta Habanos		Habanos Nordics	45.9
Skruf Strong		Skruf	36.3
Tre-Ankare White		Swedish Match	46.4
1847 Original		Philip Morris	36.8
CRP1		CORESTA Reference	44.7

**Table 6:** **United States STPs**

**US STPs**	**Style**	**Manufacturer**	**Water content (%)**
Beech Nut	Chewing tobacco	Swedish Match	21.3
Chattanooga		Swisher Int.	18.7
Durango		North Atlantic Trading Co.	20.1
Lancaster		Swisher Int.	20.2
Levi Garrett		Conwood	17.5
Morgans		Conwood	18.8
Red Man Gold		Swedish Match	21.1
Red Man Regular		Swedish Match	20.6
Southern Pride		Swedish Match	21.2
Starr		Swisher Int.	18.0
Stoker 707 Wintergreen		Swedish Match	18.7
Taylors Pride		Conwood	16.0
Trophy		Swedish Match	19.2
Bruton	Dry snuff	US Smokeless Tobacco Co.	5.8
Dental Sweet		Conwood	4.5
Garrett		Conwood	4.8
Honest		Conwood	4.3
Square		Swisher	7.4
CRP3		CORESTA Reference	6.8
Ariva Java	Hard pellet	Star Scientific	3.1
Stonewall Wintergreen		Star Scientific	2.7
Oliver Twist Original	Soft pellet	House of Oliver Twist	19.7
Copenhagen LC	Moist snuff	US Smokeless Tobacco Co.	47.1
Copenhagen Straight LC		US Smokeless Tobacco Co.	50.1
Grizzly Natural LC		Conwood	49.6
Husky Natural FC		US Smokeless Tobacco Co.	51.4
Husky Straight LC		US Smokeless Tobacco Co.	51.0
Husky Wintergreen		US Smokeless Tobacco Co.	50.3
Kayak Straight LC		Swisher	50.4
Kodiak Straight LC		Conwood	48.8
Kodiak Wintergreen		Conwood	48.0
Red Seal Natural FC		US Smokeless Tobacco Co.	49.2
Red Seal Natural LC		US Smokeless Tobacco Co.	50.1
Silver Creek		Swisher	49.5
Skoal Straight		US Smokeless Tobacco Co.	50.3
Timber Wolf Natural FC		Swedish Match	47.8
Timber Wolf Straight LC		Swedish Match	50.0
CRP2		CORESTA Reference	49.2
Cannonball	Plug	Conwood	15.4
Camel Frost	US snus	RJ Reynolds Co.	26.8
Camel Mellow		RJ Reynolds Co.	27.5
Marlboro Mild		Philip Morris	9.4
Marlboro Peppermint		Philip Morris	9.4
Marlboro Rich		Philip Morris	17.2
Marlboro Spearmint		Philip Morris	9.2

#### Reagents

Hexane (SpS grade) and technical grade methanol were obtained from Romil Ltd (Cambridge, UK). 1.0 N hydrochloric acid, 2,3,4,5,6-pentafluorobenzaldehyde (PFB) (purity 98%), anhydrous sodium sulphate (≥99.0%), acetic acid (purity ≥99.0%), and hydrazine sulphate (purity 99%) were obtained from Sigma-Aldrich (Gillingham, Dorset, UK). The calibration standard, decafluorobenzaldehyde azine (DFBA) (purity 99.8%), was prepared following the method of Liu et al. [9]. The purity of DFBA was characterised by GC/MS and Differential Scanning Calorimetry.

#### GC–MS conditions

**Figure 6:**
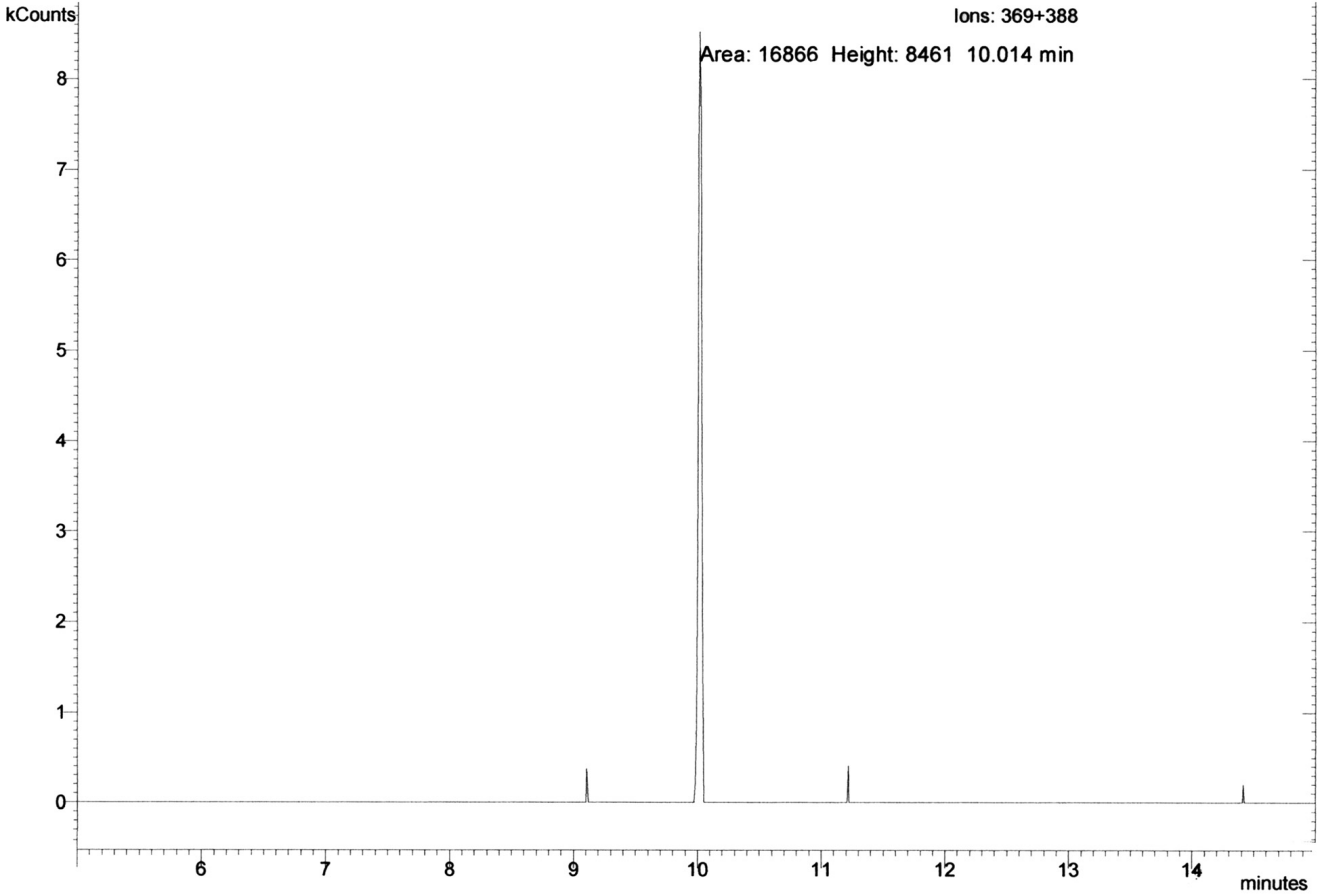
**Typical GC–MS chromatogram for a 0.5 μg/ml decafluorobenzaldehyde azine (DFBA) standard (equivalent to 0.04 μg/ml hydrazine).** Chromatogram shows signal from mass 388.

GC–MS analyses were performed using a Varian 3800–Saturn 4D GC–ion trap mass spectrometer, coupled with a Varian CP-8400 autosampler and a Saturn GC–MS Workstation running Star software version 5.51 and the following conditions: column, 30 m × 0.35 mm × 0.25 µm Zebron ZB-5 capillary column; oven program, 70°C increased to 250°C at 15°C/min, followed by a 3-min dwell time (15-min run time); injection temperature, 200°C; transfer line temperature, 220°C; manifold temperature, 250°C; injection volume, 2 µl; injection, splitless; helium flow rate, 1.0 mL/min; acquisition rate, m/z 40–550; filament delay, 5 min; quantitative ions, m/z 388 + 369 (corresponding to the molecular ion C_14_F_10_N_2_+ and a loss of F). An example chromatogram, for a standard solution of DFBA, is presented in Figure 6.

#### Preparation of standards and tobacco samples for method validation

The DFBA standard was characterized by GC–MS and the purity was determined by differential scanning calorimetry. A stock standard solution, nominally 1000 µg/mL, of DFBA in hexane, was prepared in accordance with Liu et al. [9], and diluted with hexane to give standard solutions in the range 0.20 to 2.0 µg/mL of DFBA. The solutions were tested and found to be stable at room temperature for 4 weeks.

A stock solution of hydrazine sulphate, nominally 500 µg/mL of hydrazine in deionised water, was prepared for the recovery experiments. The exact concentration of hydrazine in the stock solution was calculated using the formula:

$$ \frac{Weight\  of\  hydrazine\  sulphate\ (g)\times purity\  of\  hydrazine\  sulphate\ \left(\%\right)\times 32.05\times {10}^6\ \mu g.m{L}^{-1}}{10^4\times 130.12} $$

where 32.05/130.12 is the conversion factor for hydrazine sulphate to hydrazine. The solution was stable at room temperature for 4 weeks. Diluted solutions of 1.0 to 10 µg/mL of hydrazine in deionized water were freshly prepared for recovery experiments.

Five different types of STP were used for the validation experiments: loose snus (Ettan, Swedish Match), dry snuff (Garrett, Conwood), chewing tobacco (Taylor’s Pride, Conwood), hard pellet (Stonewall Wintergreen, Star Scientific), and plug (Day’s Work, Swedish Match). The samples were extracted in hexane as follows. Two grams of STP were added to 50 mL of 20:80 methanol:0.1 N hydrochloric acid (aq.), and the mixture was incubated at room temperature for 1 hour. For finely divided tobacco and pellet samples, the flask was placed in an ultrasonic bath for 10 min; for leafy tobacco products the mixture was macerated with a blender for 10 min. After centrifugation for 5 min at 3000 rpm, 25 mL of supernatant was added to 0.2 mL of acetic acid and 10.0 mL of 1.0% PFB in methanol, and the mixture was swirled and allowed to stand at room temperature for 1 hour. Next, 20 mL of hexane was added, and the mixture shaken for 1 min. The aqueous phase was run into a clean flask, and the hexane fraction was passed over anhydrous sodium sulphate. The aqueous phase was extracted again by the above procedure with 20 mL of hexane, and then again with 10 mL of hexane, and the three extracts were combined. The combined hexane extract was reduced to approximately 1 mL using a rotary evaporator, transferred to a 2-mL volumetric flask, and made up to 2 mL with fresh hexane, ready for application to GC–MS.

The amount of hydrazine in each tobacco sample was calculated using the formula:

$$ \frac{Test\  sample\  area \times std.\  conc.\ \left(\mu g.m{L}^{-1}\right)\times 50\times 2\times 32.05\times 1000}{Mean\ std.\  area\times 2\times 25\times 388.17} $$

where 32.05/388.17 is the conversion factor for DFBA to hydrazine. Values in this study are reported in units of ng/g, as the common portion sizes of use are gram sized [40].

For recovery experiments, 2 g of STP was added to 100 µl of a standard hydrazine solution (0.5 μg/g, 0.05 μg/g and 0.025 μg/g hydrazine). The mixture was swirled and allowed to stand at room temperature for 10 min to enable the hydrazine to penetrate the matrix. Next, 50 mL of 20:80 methanol:0.1 N hydrochloric acid (aq.) was added and the tobacco sample extracted as described above. Accuracy was determined as the mean recovery ± relative standard deviation (RSD). Precision was determined as the repeatability RSD at each level.

#### Water content

STP samples were analysed for water content using Karl Fisher Coulometric analysis with an KEM MKC-500 analyser (Kyoto Electronics, Tokyo, Japan). Approximately 2 g STP was weighed into a 25 ml snap-top vial. 20.0 ml MeOH was added and the sample sonicated for 15 minutes before being allowed to steep and settle for at least 2 hours. 100 μl of methanol was sampled and injected into the Karl Fisher analysis cell. Water blanks were subtracted, and analyses conducted in triplicate.

## Conclusions

In this study we have developed and validated a method to determine levels of hydrazine in tobacco, using PFB as a complexing agent to form the azine, DFBA, which was then quantified by GC–MS. In a series of 74 brands of smokeless tobacco from the US and Sweden covering all major product styles - snus, chewing tobacco, moist snuff, dry snuff, plug and pellet products - hydrazine concentrations were all below the level of quantification of 26.5 ng/g product. Peaks consistent with hydrazine were identified, at trace levels (below limit of detection), in the chromatograms of less than half of the 74 STPs, but at levels considerably lower than the only previous study to have quantified hydrazine in tobacco. All previous references to the presence of hydrazine in tobacco and STPs are based on a single study from 1974 [9] which measured the hydrazine contents of tobacco from five different cigarettes (4 experimental and 1 commercial).

There is insufficient information to explain the differences in results between the present study and the earlier study. However the much lower levels of hydrazine found in the present study are consistent with the reductions in maximum levels of hydrazine residues permitted in MH, which were legislated subsequent to the original study of Liu et al. Also our method used shorter times for complexation of hydrazine residues with PFB. The shorter complexation time may have minimised artifactual formation of DFBA through complexation with non-hydrazine moieties such as hydrazones and azines as Liu et al. reported as probable in their study. Nevertheless, the current study demonstrates that the presence of hydrazine in contemporary STPs is relatively infrequent, and when present hydrazine is at levels below the limits of quantification and detection that can be achieved using current analytical best-practice.

## Abbreviations

BLD: Below the limit of detection
CORESTA: Cooperation centre for scientific research relative to tobacco
DFBA: Decafluorobenzaldehyde azine
FDA: US food and drug administration
GC-MS: Gas chromatography - mass spectrometry
GRL: Guidance residue level
HPHC: Harmful and potentially harmful constituents
IARC: International agency for research in cancer
LOD: Limit of detection
LOQ: Limit of quantification
MH: Maleic hydrazide
PFB: Pentafluorobenzaldehyde
PFBA: Pentafluorobenzaldehyde azine
RSD: Relative standard deviation
STP: Smokeless tobacco product
